# Manufacturing Scalable Carbon Nanotube–Silicone/Kevlar Fabrics

**DOI:** 10.3390/nano13192728

**Published:** 2023-10-08

**Authors:** Prakash Giri, Vamsi Krishna Reddy Kondapalli, Kavitha Mulackampilly Joseph, Vesselin Shanov, Mark Schulz

**Affiliations:** 1 Department of Mechanical and Materials Engineering, University of Cincinnati, Cincinnati, OH 45221, USA; kondapvy@mail.uc.edu (V.K.R.K.); josephka@mail.uc.edu (K.M.J.); shanovvn@ucmail.uc.edu (V.S.); 2 Department of Chemical and Environmental Engineering, University of Cincinnati, Cincinnati, OH 45221, USA

**Keywords:** CNT-silicone, CNT sheets, Kevlar yarn, Kevlar fabric, Kevlar veil

## Abstract

Carbon nanotube (CNT) hybrid composites were formed by combining a CNT and silicone elastomer solution with Kevlar yarn, Kevlar fabric, and Kevlar veil materials. The integration of a CNT-silicone matrix with Kevlar yarn and fabric materials produced a composite with moderate electrical and thermal conductivity due to CNT fabric combined with the strength of Kevlar fabric or yarn. In the material synthesis, a notable difficulty was that the CNT-silicone did not bond strongly to the Kevlar. The composites passed the Vertical Flame Test ASTM D6413 and the Forced Air Oven Test NFPA 1971. These hybrid composites can have multiple applications in areas requiring favorable conductivity, strength, and flame and heat resistance. The application areas include firefighter apparel, military equipment, conductive/smart structures, and flexible electronics. The synthesis process used to manufacture CNT-silicone/Kevlar composites yielded composite sheets with an area of 2250 cm^2^. The process is scalable and customizable for the synthesis of CNT composites with tailored properties. Improvements in the bonding of CNT-silicone to Kevlar are being investigated.

## 1. Introduction

**Figure 1:**
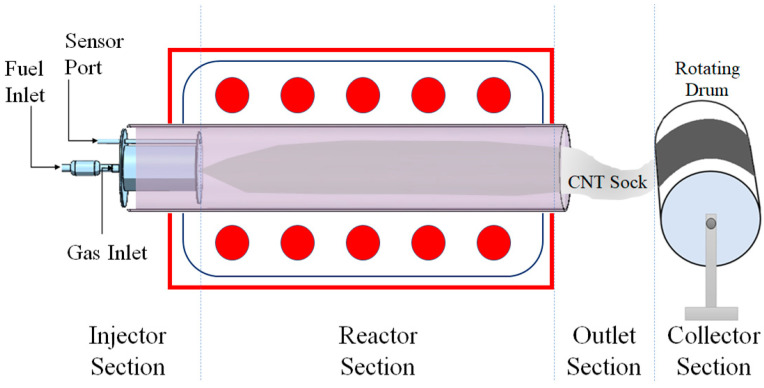
The floating catalyst chemical vapor deposition (FCCVD) for CNT sheet synthesis (also called a floating catalyst reactor system).

Carbon nanotubes (CNTs) are 1D nanostructures composed of single carbon atoms with unique C-C covalent bonding and a seamless hexagonal network [1]. CNTs have been attracting the interest of researchers due to their important properties such as high mechanical strength and low density, high aspect ratio, favorable electrical and thermal conductivity, flame resistance, unique optical properties, semiconducting behavior, and so on [2,3,4,5]. Arc discharge, laser ablation, gas phase pyrolysis (Figure 1), a bottom-up organic approach, and chemical vapor deposition (CVD) are some of the well-known methods used for CNT synthesis [6].

The exact growth mechanism of CNTs is still debated, but the CVD method of CNT synthesis is popular among researchers as CVD can be conveniently used to tailor CNT properties such as diameter, orientation, conductivity, porosity, permeability, and functionalization, thus expanding their use to a wide range of applications [6,7]. Thermal or plasma-enhanced catalytic CVD, water or oxygen-assisted CVD, hot-filament CVD, microwave plasma CVD, and radio frequency CVD are some of the CVD techniques used for large-scale CNT production [6,8,9,10,11,12,13].

Nanoelectronics, nanocomposites, energy storage, hydrogen storage, biomedicine, wearable electronics, smart materials and sensors, air/water filtration, and drug delivery are some, but not all, application areas of CNT materials [2,14]. However, there are some challenges to achieving quick and widespread use. Although CNTs are customizable, it is difficult to maintain precise conditions to tune their properties and gain control over nanotube growth [15,16]. Every parameter, such as the type and dimensions of the CVD equipment, reaction temperature, reaction gas and gas flow rates, the selection of catalyst, etc., needs to be considered for the synthesis of CNTs with tailored and reproducible properties [17,18]. Macroscale and low-cost synthesis of individual CNTs is still in the developing phase, and it is difficult to extrapolate their nanoscale properties to macroscale material forms [19].

Over the years, CNT composites with different polymers, metals, and ceramics have been developed for different applications, including aerospace, biomedicine, electromagnetic interference shielding, supercapacitors, functional textiles, and many more [20,21,22,23,24,25,26]. Studies have also demonstrated the capacity of CNT composites to improve friction and wear behavior in equipment and parts [27,28,29]. With the improved synthesis procedures, researchers have been able to synthesize large-scale CNT composites, including pristine CNT sheets and tapes [30,31,32,33,34,35]. CNT composites with cotton and spandex have been studied for dyeable, wearable, and shielding applications [30,36,37]. Researchers have shown that some application areas of CNT/Kevlar composites improve ballistic performance, wearable electronics, fabrics with anti-impact properties, textiles with sensing capabilities, and triboelectric nanogenerators [38,39,40]. The CNT/Kevlar composites for those applications were developed by soaking pieces of Kevlar fabric in CNT using ultrasonic dispersion and impregnation methods [38,39,40]. To our knowledge, macroscale and continuous synthesis of CNT/Kevlar composites have not yet been reported.

Kevlar fabrics have high strength, high modulus, and high impact resistance, and they can withstand high temperatures [41,42,43]. The new developments in CNT/Kevlar composite fabrics provide excellent composite strength, modest electrical conductivity, and high flame resistance. There can be a wide range of applications for such textiles in the areas of defense, automobile/aircraft structures and components, functional textiles, firefighting and insulation, etc. They can also be useful for printable electronics and thermal management of electronic devices. Looking at the applicational opportunities that CNT/Kevlar composites could provide, we have developed three variations of composites: CNT-silicone/Kevlar yarn, CNT-silicone/Kevlar fabric, and CNT-silicone/Kevlar veil.

## 2. Synthesis of Macroscale CNT Sheet

The pristine CNT sheet was synthesized on a horizontal floating catalyst chemical vapor deposition (FCCVD) reactor, as shown in Figure 1. The reactor has an injector for the controlled flow of gases (ultra-high-purity argon and hydrogen). The gas mixture carries CNT through the reactor tube to the glove box. The injector also contains temperature sensors and a passage for fuel.

Fuel consisting of methanol (Fisher Chemical, Waltham, MA, USA), ferrocene (Fisher Chemical), n-hexane (Lab Alley, Spicewood, TX, USA), and thiophene (Aldrich Chemistry, St. Louis, MO, USA) was prepared and fed into the reactor at a rate of 90 mL/h through an atomizer, the role of which is to disperse a catalyst precursor into the reactor chamber for CNT growth. The carrier gases, argon and hydrogen from the inlet assist in dispersing the catalyst and fuel inside the reactor. The flow rate of the fuel was controlled with the help of a syringe pump.

**Figure 2:**
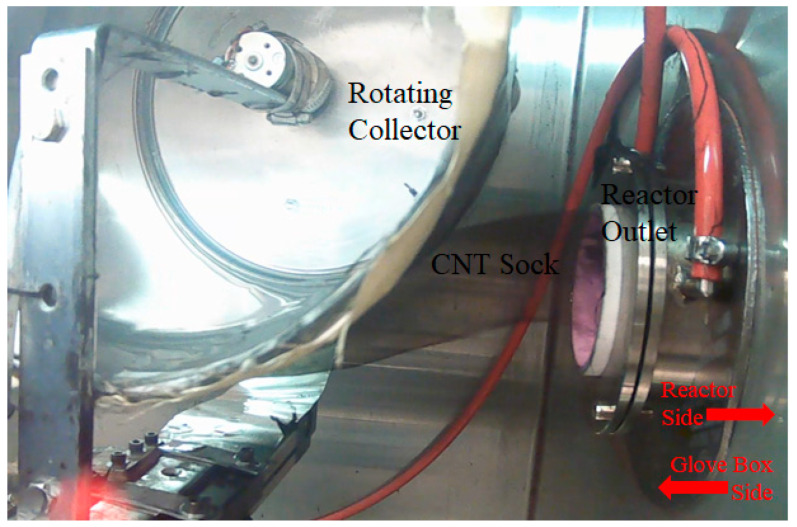
The collection of CNT sock coming out of the FCCVD reactor.

The temperature of the reactor was maintained at 1250 °C for the entire synthesis duration. In an inert atmosphere of argon inside a glove box, a drum collector, rotating at a speed of 4.3 rpm, was positioned 5 cm from the reactor outlet to collect the CNT sock, as shown in Figure 2. The drum also transfers the collected CNT materials around its circumference. The synthesis process can be visualized in Supplementary Material S1.

**Figure 3:**
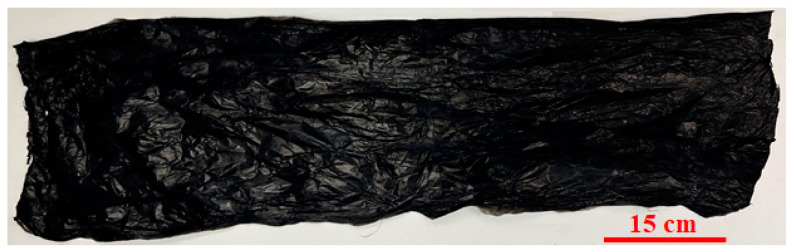
Pristine CNT sheet.

Over time, a smooth film of CNTs was collected. For pristine CNTs, acetone was used for the densification of the sock collected. Using this process, a typical synthesis experiment ranging from ~90 min to ~120 min can form a CNT sheet of ~20 microns in thickness. The area of the sheet thus formed is 2250 cm^2^. Figure 3 presents a pristine CNT sheet formed using the process. The sheet had a length of 90 cm and a width of 25 cm. The thickness of the sheet was 20 microns.

## 3. Synthesis of CNT-Silicone Composite Sheets

**Figure 4:**
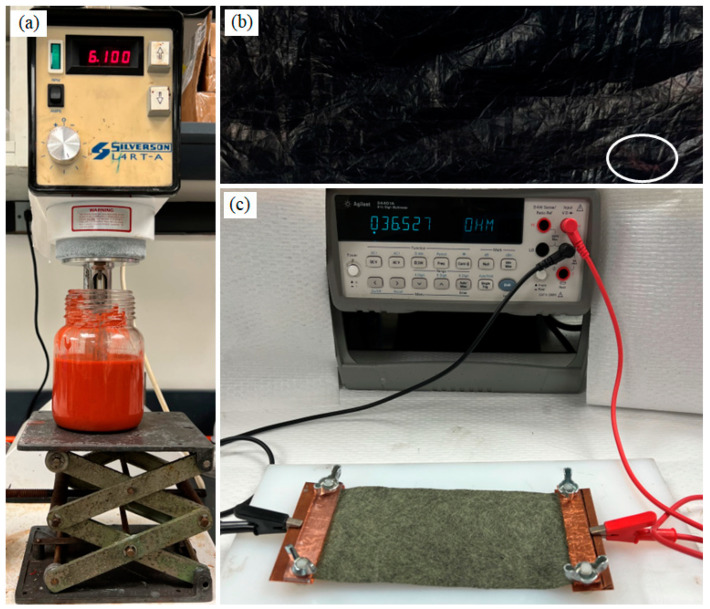
Manufacturing and testing CNT silicone sheets: (**a**) the shear mixture to prepare silicone and naphthalene solution; (**b**) the CNT-silicone sheet produced; (**c**) the two-point method of resistivity measurement.

CNT-silicone composite sheets were synthesized by densifying the CNT sock with a silicone solution. Red RTV Gasket Maker High-Temperature Silicone (Permatex, Solon, OH, USA) was dissolved in naphthalene at different concentrations to prepare a CNT-silicone solution. For a typical synthesis experiment that forms a uniform silicone solution over time, a 0.03 g/mL solution of high-temperature silicone elastomer was prepared in naphthalene with the help of a shear mixer (Silverson L4RT-A, East Longmeadow, MA, USA), as shown in Figure 4a. A section of CNT-silicone sheet formed by the densification of the CNT sock by the silicone-naphthalene solution is shown in Figure 4b. The red coloration, which is due to the deposition of the red silicone solution, can be seen in some sections of the CNT-silicone composite sheet (indicated by the white oval). The CNT-silicone composite sheets were more handleable than the pristine CNT sheets.

Except for the thickness, the dimensions of the CNT-silicone sheets were the same as the pristine CNT sheets, i.e., 90 cm × 25 cm. The thickness increased from 20 microns for the pristine sheet to ~22 microns for the CNT-silicone sheet. The density of the pristine CNT sheet was 0.25 g/cc, whereas the density of the CNT-silicon composite was 0.56 g/cc.

**Figure 5:**
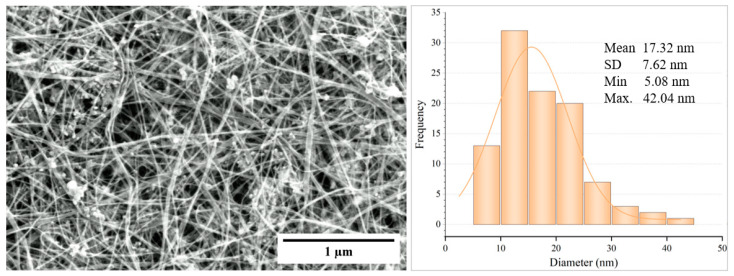
A SEM image of a pristine CNT sheet and a histogram of the strand diameters.

The microstructure of the pristine CNT sheets and the various composite sheets was analyzed using scanning electron microscopy (FEI Aprio LV-SEM, Waltham, MA, USA). The pristine CNT sheets consisted of CNT strands with diameters ranging from 5.08 nm to 42.04 nm, with a mean of 17.32 nm and a standard deviation of 7.62 nm, as shown in Figure 5.

**Figure 6:**
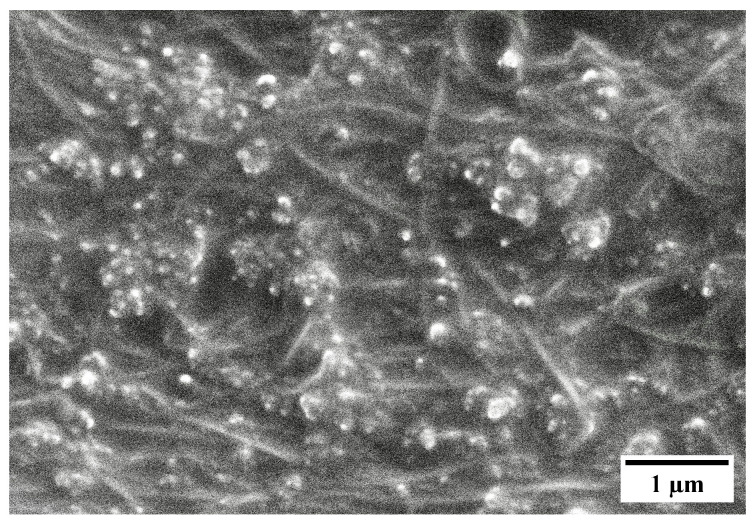
A SEM image of a CNT-silicone composite sheet.

Individual CNT strands were not observed in the SEM imaging of CNT-silicone samples, Figure 6. The reason for this was the presence of a thin silicone film surrounding the CNT strands, a result of the densification step, and though this film was present, the CNT-silicone composite still possessed conductive properties. Figure 5 and Figure 6 are also typical SEM images for the CNT/Kevlar and CNT-silicone/Kevlar composites because pristine CNT and CNT-silicone from a similar synthesis process were deposited on top of the Kevlar fabrics.

The resistivity of both membranes was calculated using the two-probe method, as shown in Figure 4c. The resistivity was given as [1,44]:

(1) $$\rho =\frac{\mathrm{RA}}{L},$$

Here, ρ is the resistivity, L is the length of the CNT sheet, A is the cross-sectional area of the sheet, and R is the resistance measured across the length of the sheet. Resistivity values are given in Table 1.

The conductivity anisotropy ratio observed was a result of a higher number of junctions (i.e., fewer aligning CNTs) along the direction orthogonal to that of the sock collection [45]. Similarly, the electrical resistance of the sheet would be much greater through the thickness of the sheet than in the plane. The greater resistance through the thickness is due to the nanotube-to-nanotube lateral junctions.

The tensile strength of the pristine CNT sheet and the CNT-silicone sheet was measured using a Micro Instron Testing Machine, Model 5948. The sample specimens with a gauge length of 20 mm and a width of 2 mm were prepared and supported with the assistance of a rectangular paper specimen holder. Pneumatic grips were used for the test.

**Figure 7:**
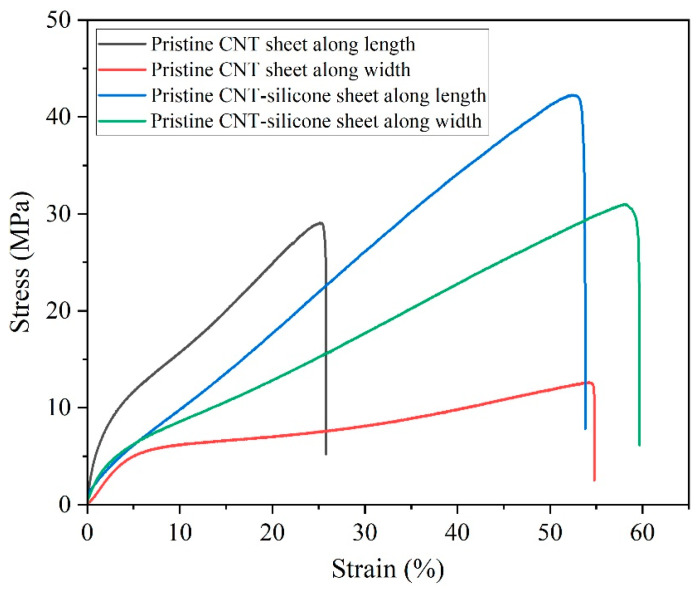
Stress-strain plots of the pristine CNT sheet and the CNT-silicone sheet.

The pristine CNT sheet had a maximum tensile strength of 29 MPa at 25% strain along the length (synthesis direction). A maximum tensile strength of 12.5 MPa was observed at 53% strain along the width (the direction perpendicular to the synthesis direction). The decrease in strength but increase in strain was a result of anisotropy generated during the synthesis process. A greater number of CNT junctions were present along the width of the sample, and they tended to separate when subjected to stress, causing higher strain but lower strength (Figure 7) [45].

The CNT-silicone sheet showed a strength of 42.2 MPa at 52.5% strain along the length. The same sample along the width was 31 MPa at 58% strain. An improvement of 1.5 times in strength and 2.1 times in strain was obtained along the length due to the integration of silicone elastomer. Similarly, an improvement of 2.5 times in strength and 1.1 times in strain was obtained along the width. From these results, it can be concluded that the use of silicone elastomer improves the strength and strain of CNT sheets and tends to make the sheets more isotropic in terms of their strength.

**Figure 8:**
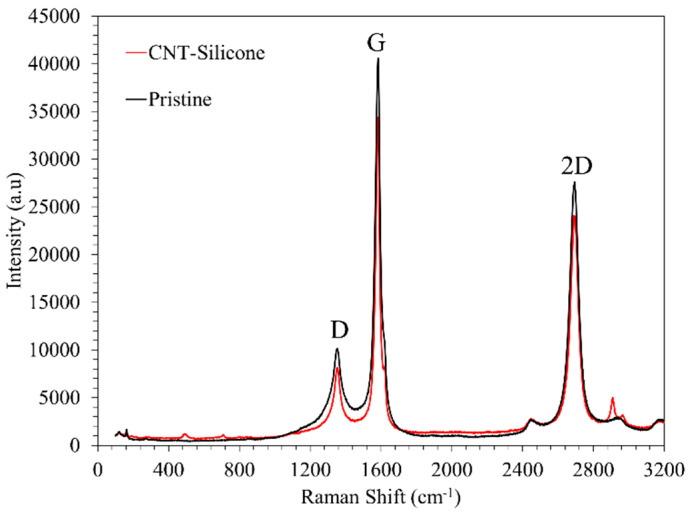
The Raman spectra of the CNT sheet and the CNT-silicone sheet highlight three signature peaks.

Renishaw inVia Raman spectroscopy with a 514 nm wavelength was used to analyze the CNT-based materials. A laser spot size of ~1 µm^2^ and a lens of 50× magnification were used for this study. The exposure time (10 s) and the number of accumulations (3) were kept the same for all the samples. The Raman spectra of the CNT sheet and CNT-silicone sheet are shown in Figure 8. Both spectra show the signature peaks of CNT, i.e., D, G, and 2D peaks.

**Figure 9:**
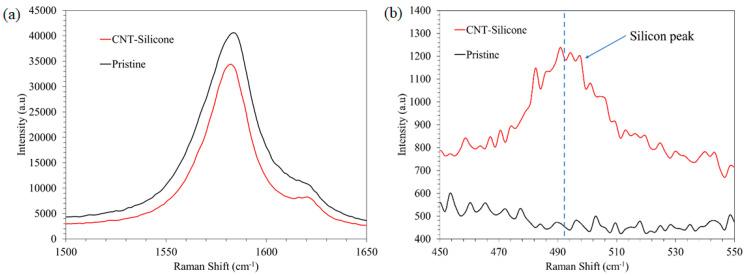
(**a**) The Raman G peak of the CNT sheet and the CNT-silicone sheet; (**b**) the silicone peak of the CNT sheet and the CNT-silicone sheet.

The G peaks of both materials are shown in Figure 9a. No change in the G peak position or I(D)/I(G) was observed between the pristine CNT sheet and the CNT-silicone sheet, highlighting that there were no stress/damage/defects in the CNTs due to the addition of silicone [46].

Furthermore, a silicone peak of around 500 cm^−1^ was observed in the CNT-silicone Raman spectra indicating the presence of silicone in the CNT-silicone sheets, as shown in Figure 9b. This peak was absent in the pristine CNT peak, as seen in Figure 9b.

## 4. Synthesis of CNT-Silicone/Kevlar Composites

The synthesis of CNT-silicone/Kevlar composites was performed by continuously collecting CNT sock with periodic, simultaneous densification using a silicone solution in the harvest box of a FCCVD reactor. Three types of Kevlar textiles were used for our synthesis process: Kevlar yarn, Kevlar plane weave fabric, and Kevlar veil.

### 4.1. CNT-Silicone/Kevlar Yarn Composite

**Figure 10:**
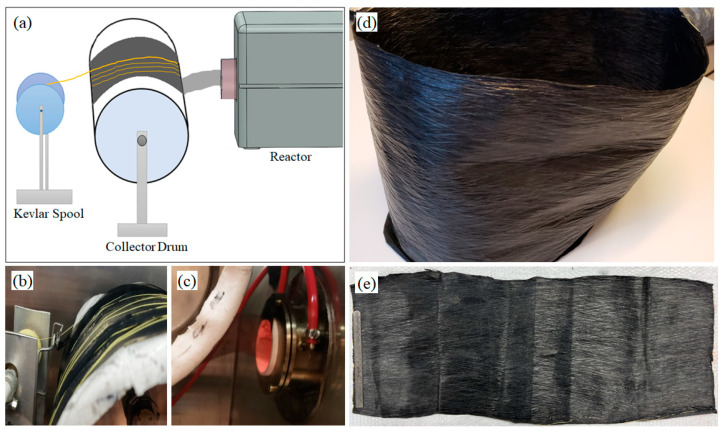
Manufacturing CNT-silicone/Kevlar yarn composites: (**a**) schematic of the CNT-silicone/Kevlar yarn sheet synthesis process; (**b**) the Kevlar yarn collection side of the collector drum; (**c**) the CNT-sock collection side of the collector drum; (**d**) the CNT-silicone/Kevlar yarn composite sheet as taken out of the collector drum; (**e**) the CNT-silicone/Kevlar yarn composite sheet.

A CNT-silicone/Kevlar yarn hybrid composite sheet was synthesized to utilize the high strength of Kevlar yarn in CNT composites. A spool of Kevlar yarn was taken and allowed to wind onto the CNT-collector drum in a fashion similar to the CNT sock collection. Three major steps were involved: (a) CNT sock exiting the reactor outlet was collected in the rotating drum; (b) Kevlar yarn unwinding from a spool was collected on the rotating drum simultaneously, in addition to the CNT sock, but coming from the opposite direction; and (c) the CNT sock was densified periodically by spraying a silicone-naphthalene solution. A typical synthesis process took about two hours (Figure 10).

**Figure 11:**
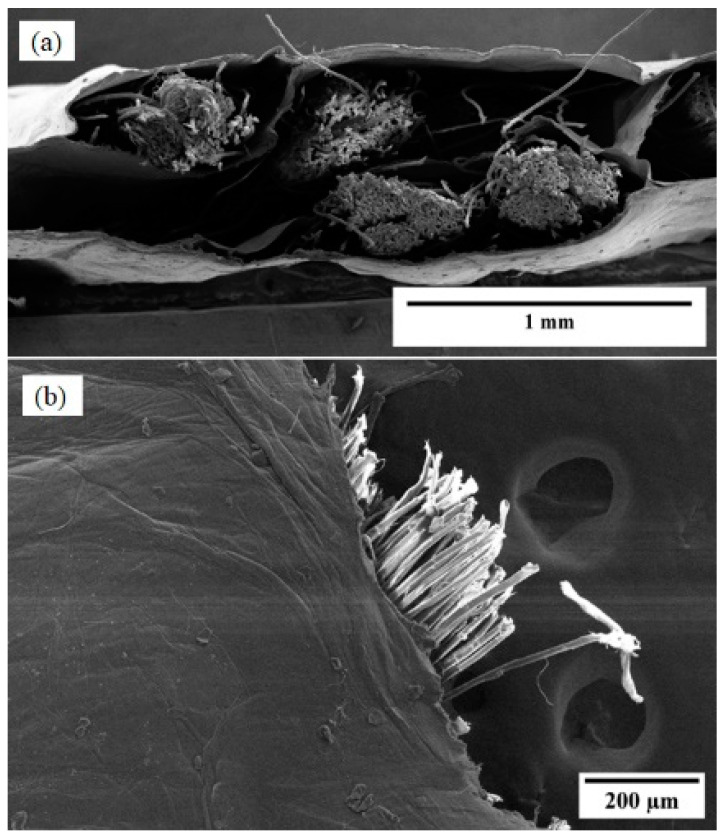
CNT-silicone/Kevlar yarn fabric: (**a**) cross-section; (**b**) edge section of the CNT-silicone/Kevlar yarn composite.

The Kevlar yarn extends along the length of the composite sheet that has a dimension of 90 cm × 25 cm as shown in Figure 10e. Figure 11a presents the cross-sectional features of the CNT-silicone/Kevlar yarn composite, where we can observe the reinforcement of Kevlar yarn surrounded by the CNT-silicone matrix. Figure 11b shows the edge of the composite.

The average thickness of the CNT-silicone/Kevlar yarn composite was 435 microns. The Kevlar yarn was continuously wrapped a few hundred times on the CNT sheet. The video of the synthesis process is shown In Supplementary Material S2.

### 4.2. CNT-Silicone/Kevlar Fabric Composite

**Figure 12:**
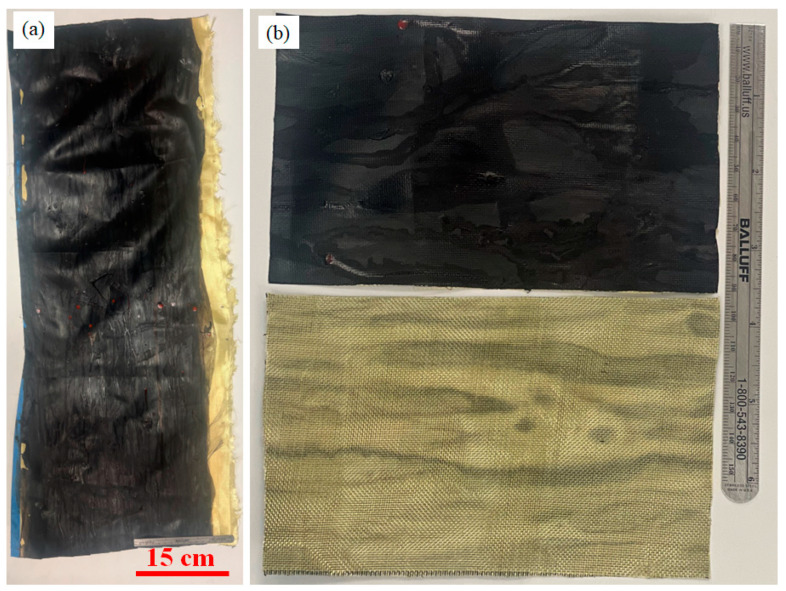
CNT-silicone/Kevlar fabric images without magnification: (**a**) macroscale CNT-silicone/Kevlar fabric composite; (**b**) sections of the front and back surfaces of the fabric.

Kevlar fabric (Kevlar Plain Weave 195d 38″/96.52 cm 1.7 oz/58 gsm, Composite Envisions, WI, USA) was wound around the rotating collector, covering it. The CNT sock coming out of the reactor was collected on the top of the fabric and periodically densified with a 0.03 g/mL solution of silicone in naphthalene. The Kevlar fabric improved the handleability and formed a CNT-silicone/Kevlar fabric hybrid composite textile, as shown in Figure 12. The bonding of the CNT-silicone matrix material to the Kevlar fabric was favorable in this case.

**Figure 13:**
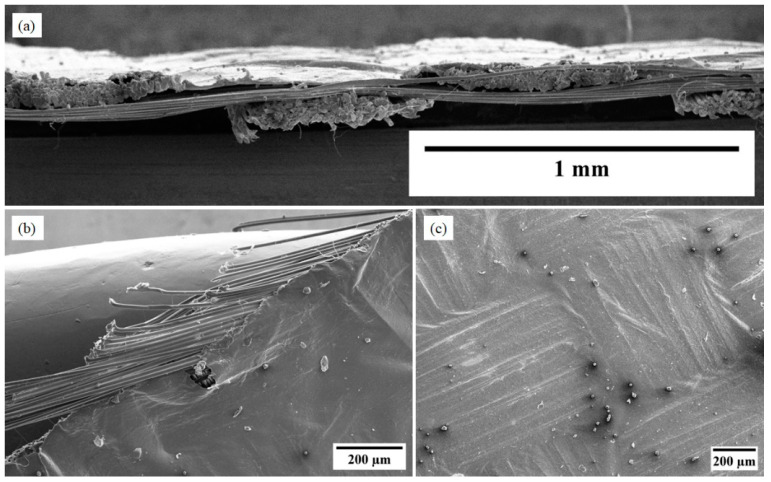
CNT-silicone/Kevlar fabric SEM images: (**a**) cross-section of CNT-silicone/Kevlar fabric composite; (**b**) edge of CNT-silicone/Kevlar fabric composite; (**c**) patterns seen in the composite as a result of patterns in the underlying fabric.

The cross-section of the CNT-silicone/Kevlar fabric composite is shown in Figure 13a. Figure 13b shows the edge of the composite, showing the deposition of CNT-silicone on the fabric fibers. Patterns from the weaving of Kevlar fabric on the composite were also observed in the imaging, as shown in Figure 13c. This process presents a possibility for tailoring underlying fabric materials with different patterns to meet the needs of the various technical and aesthetic application areas. The thickness of the CNT-silicone film can affect the pattern’s appearance. These composites can also be manufactured in the required shapes, as they are durable, flexible, easy to cut, and can be sewn like fabrics.

Different weights of Kevlar 29 and Kevlar 49 fabrics were used to form the textile composites. The CNT-silicone bonded to some of the Kevlar fabrics and did not bond to others. The bonding is being investigated as further work. Sizing on the Kevlar fabric and curing parameters may have affected the bonding.

### 4.3. CNT-Silicone/Kevlar Veil Composite

**Figure 14:**
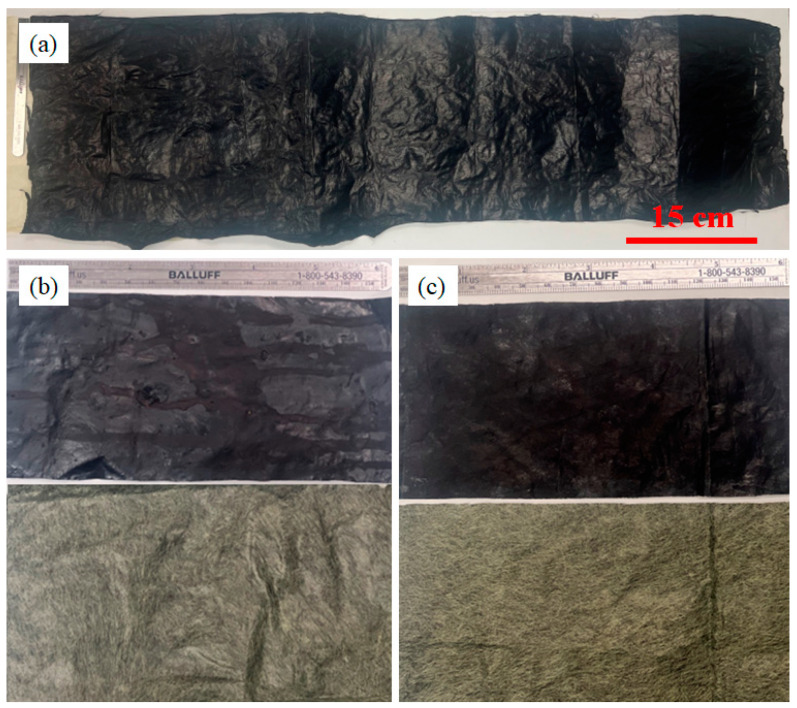
CNT-silicone/Kevlar veil fabric: (**a**) macroscale CNT/Kevlar veil composite; (**b**) front and back sections of CNT-silicone/Kevlar veil composite; (**c**) front and back sections of CNT/Kevlar veil composite.

The Kevlar veil (Kevlar Fabric Veil Chopped Mat 35.5″/90.17 cm, 26 oz/8 gsm, Composite Envisions, WI, USA) was wound around the rotating collector as stated in Section 4.2. The CNT sock exiting the reactor was collected on top of the veil with periodic densification by a silicone-naphthalene solution for about 25 min to generate a thin CNT/Kevlar veil composite. Since the veil material might be used for filter membranes, another synthesis was carried out by collecting CNT sock with densification by acetone on the top of the veil material for about 25 min. Densification with acetone instead of silicone solution helps obtain a more porous CNT hybrid textile that could be used for various permeability applications. The veil materials thus manufactured are shown in Figure 14.

**Figure 15:**
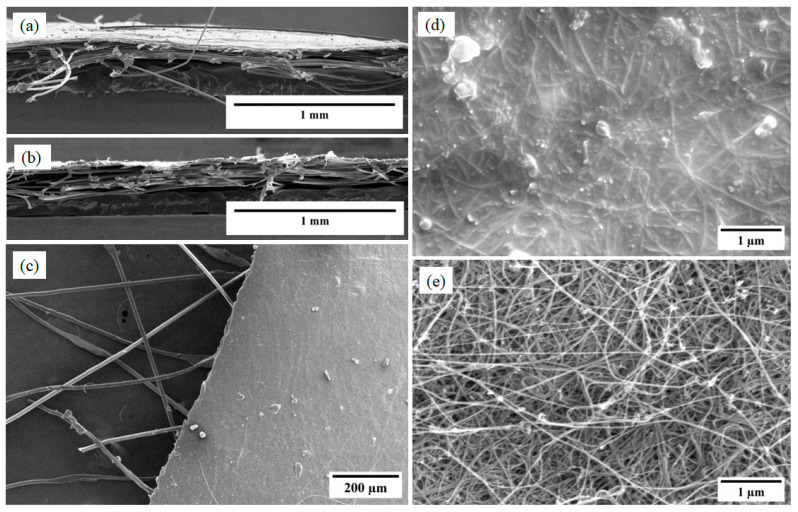
CNT Kevlar veil materials: (**a**,**b**) cross-section of CNT-silicone/Kevlar veil and CNT/Kevlar veil composites; (**c**) edge of CNT-silicone/Kevlar veil composite; (**d**) SEM of CNT-silicone/Kevlar veil composite; (**e**) SEM of CNT/Kevlar veil composite.

The cross-sectional features of the CNT-silicone/Kevlar veil and the CNT/Kevlar veil can be seen in Figure 15a,b, respectively. Figure 15c presents the edge of the composite, where Kevlar veil fibers are sparsely distributed to reinforce the composites. Figure 15d,e is similar to those presented for CNT-silicone and pristine CNT sheets.

## 5. Results and Discussions

Macroscale pristine CNT, CNT-silicone, CNT-silicone/Kevlar yarn, CNT-silicone/Kevlar fabric, CNT-silicone/Kevlar veil, and CNT/Kevlar veil composites were synthesized using a scalable FCCVD system. The length and width of the sheets were 90 cm by 25 cm. The thickness and density of the pristine CNT sheets and the CNT-silicone sheets were 20 microns, 0.25 g/cc, and 22 microns, 0.56 g/cc, respectively, as discussed in Section 3. The thickness of CNT-silicone/Kevlar yarn composites was 435 microns, and the CNT-silicone/Kevlar fabric was 116 microns. The CNT-silicone/Kevlar veil composites for this synthesis were measured at 95 microns in thickness, whereas the CNT/Kevlar veil was 90 microns. The thickness of these membranes can be varied easily by modifying the collection period of the sock. The length and width of the sheets can also be altered using different parameters (a larger drum diameter) for the collector setup.

Pristine CNT sheets, CNT-silicone composite sheets, and CNT-silicone/Kevlar yarn sheets were thermally and electrically conductive along their front and back surfaces, whereas CNT-silicone/Kevlar fabric composites were conductive on the surface with CNT-silicone deposition. Interestingly, CNT-silicone/Kevlar veil and CNT/Kevlar veil composites are conductive on the surface with the CNT-silicone deposition, whereas the conductivity on the other surface can depend upon the contact pressure applied. This property is being studied for impact and damage detection in sensitive equipment and machinery, and it is part of follow-up work.

The density, resistivity, and anisotropy ratios of the membranes are tabulated in Table 2.

**Figure 16:**
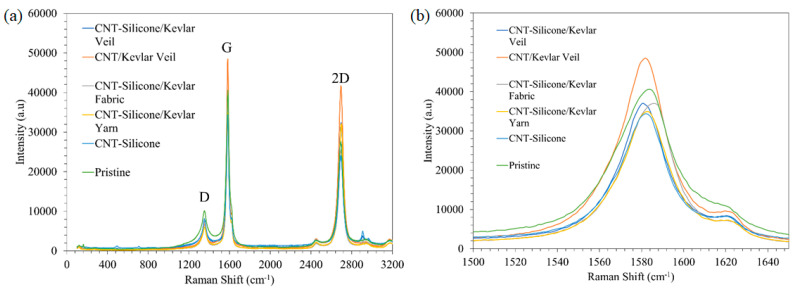
(**a**) Raman spectra of CNT sheet and CNT-silicone sheet; (**b**) Raman G peak of CNT-silicone/Kevlar veil, CNT/Kevlar veil, CNT-silicone/Kevlar fabric, CNT-silicone/Kevlar yarn, CNT-silicone, and Pristine CNT.

The Raman spectra of CNT-silicone/Kevlar veil, CNT/Kevlar, CNT-silicone/Kevlar fabric, CNT-silicone/Kevlar yarn, CNT-silicone, and pristine CNT are shown as a stack in Figure 16a. From the spectra, it is clear that neither the addition of silicone nor Kevlar made significant changes, as no change in I(D)/I(G) or the position of the G peaks shown in Figure 16b was observed. Although a slight difference in the G peak position can be seen, it is between 1580 cm^−1^ and 1590 cm^−1^, which is very common for CNTs.

**Figure 17:**
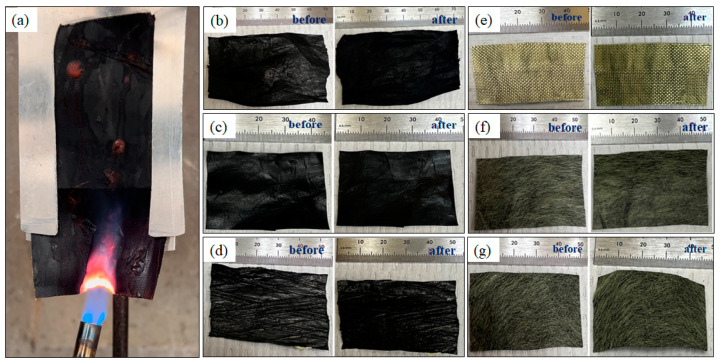
(**a**) Vertical Flame test on a CNT-silicone/Kevlar fabric sample; (**b**–**g**) Pristine CNT, CNT-silicone, CNT-silicone/Kevlar yarn, CNT-silicone/Kevlar fabric, CNT-silicone/Kevlar veil, and CNT/Kevlar veil samples before and after Forced Air Oven Test. None of the samples showed excessive char length during the Vertical Flame test. None of the samples melted or dripped during both tests. There was no noticeable shrinkage in the samples during the Forced Air Oven Test.

Pristine CNT sheets, along with other composite sheets, were thermally conductive and flame-resistant. The composites passed the Vertical Flame Test ASTM D6413/D6523M-15 and the Forced Air Oven Test NFPA 1971 [47,48]. The flame application time for vertical flame testing the samples was 12 s. All the samples exhibited less than 1 s of after-flame and after-glow time. The char length in the samples was less than 4 cm, and no melting or dripping was observed (Figure 17a). For NPFA compliance, the samples were placed in an oven at 260 °C for 5 min. All the samples remained intact without melting, dripping, or shrinking as shown in Figure 17b–g.

## 6. Safety Concerns

The manufacturing process (CNT synthesis and coating) described in this paper produces a complex mixture of flammable gases. Any oxygen leakage into the reactor system at such a high temperature (1250 °C) could result in explosions with severe consequences. An inert atmosphere for pyrolysis must be maintained in the reactor tube during the synthesis process. An oxygen sensor should be used to verify that the oxygen content in the harvest box is low. Continuous dilution of the exhaust gases with a high flow rate of fresh Argon gas or another inert gas is required to cool and reduce the concentration of the flammable gases in the harvest box. The ceramic process tube must be heated and cooled slowly to prevent cracking. Two key improvements in safety for this process are being implemented and will be reported in future publications.

## 7. Conclusions

Macroscale CNT-silicone/Kevlar composites were manufactured by reinforcing a CNT-silicone matrix with Kevlar yarns, fabrics, and veil materials. The hybrid composites are flexible, conductive, and flame-resistant. Synthesis of CNT-silicone membranes with Kevlar takes advantage of the pristine strength of Kevlar veil, fabrics, and yarns while utilizing the multi-functional properties of CNT and CNT-silicone composites. The strength of Kevlar combined with CNT and silicone properties may be beneficial in applications requiring flexibility, strength, and flame resistance, such as personal protective equipment for firefighters and first responders. Composites such as CNT-silicone/Kevlar fabric are conductive on one surface while being insulative on the opposite surface. This feature is an important requirement for a variety of applications, such as one-sided insulation, smart structures, flexible conductors, and electromagnetic shields. The thermal conductivity of these composites can also be beneficial in extending their use for thermal management of electronic devices, where their thin fabrics or membranes can act as structural support for distributing the generated heat across the surface for cooling. The Kevlar veil has a thin and sparse arrangement of tiny, chopped fibers on which the CNT-silicone matrix is layered. The veil improves the strength and handleability of the CNT-silicone sheet. The porous nature of the CNT/Kevlar veil can be used for air/water filtration. In addition, the composites with Kevlar veil materials are conductive on one surface and show conductive or insulating behavior, depending on the applied contact pressure, on the another surface. This feature is beneficial for smart structures requiring impact measurement and protection. The application areas of the CNT-silicone/Kevlar composites will be explored further in follow-up work. To summarize, the macroscale and scalable production of pristine CNT, CNT-silicone, and CNT-silicone/Kevlar composites broadens the conventional application areas of CNTs and addresses the scaling-up difficulties. One area for improvement is to increase the bonding strength of CNT-silicone to Kevlar fabric.

## Figures and Tables

**Table 1:** Resistivity and conductivity anisotropy ratios. Resistivity measurements are in the plane of the sheet. Contact resistance is included in the resistivity measurements.

CNT Sheet Type	Resistivity (Ω·cm)		Anisotropy Ratio k‖/k┴
	Along Length	Along Width	
Pristine CNT sheet	0.0043	0.0091	2.12
CNT-silicone composite sheet	0.0059	0.0163	2.76

**Table 2:** Density, resistivity, and conductivity anisotropy ratios. The data represents in-plane properties. The results include contact resistance.

CNT Sheet Type	Density (g/cc)	Resistivity (Ω·cm)		Anisotropy Ratio, k‖/k┴
		Along Length	Along Width	
CNT-silicone/Kevlar yarn	0.20	0.06	0.11	1.83
CNT-silicone/Kevlar fabric	0.90	0.11	0.26	2.36
CNT-silicone/Kevlar veil	0.30	0.09	0.09	1.89
CNT/Kevlar veil	0.17	0.06	0.06	1.67

## Data Availability

Limited data are available upon request to the corresponding authors.

## Supplementary Materials

The following supporting information can be downloaded at: https://www.mdpi.com/article/10.3390/nano13192728/s1, Video S1: CNT Sheet Synthesis; Video S2: Synthesis of CNT-Silicone/Kevlar Yarn Composite.
